# Oxaliplatin-Induced Pulmonary Fibrosis: A Rare but Fatal Reality

**DOI:** 10.7759/cureus.51411

**Published:** 2023-12-31

**Authors:** Kinnera Sahithi Urlapu, Dmitry Lvovsky

**Affiliations:** 1 Pulmonary Medicine, BronxCare Health System, Bronx, USA; 2 Pulmonary and Critical Care, BronxCare Health System, Bronx, USA

**Keywords:** chemotherapy side effects, pulmonary fibrosis, folfox, progressive ild, steroid therapy, drug-induced pulmonary toxicity, drug induced interstitial lung disease, interstitial lung disease, oxaliplatin

## Abstract

Oxaliplatin is a commonly used chemotherapy drug for the treatment of gastrointestinal malignancies, but it can lead to various side effects, including interstitial lung disease (ILD), a rare but potentially fatal condition. ILD is an inflammatory and fibrotic lung disease that can cause progressive lung damage and respiratory failure. The exact mechanism by which oxaliplatin induces ILD is not known, but it is believed to be due to an immune-mediated response, or direct toxicity via oxidative stress. The symptoms of oxaliplatin-induced ILD include cough, shortness of breath, fatigue, and weight loss. Diagnosis of oxaliplatin-induced ILD requires a high index of suspicion, and imaging tests such as chest X-rays and CT scans are used to confirm the diagnosis. Treatment options for oxaliplatin-induced ILD include corticosteroids, oxygen therapy, and early cessation of oxaliplatin therapy. Early detection and prompt management are crucial to improve the prognosis of patients with oxaliplatin-induced ILD.

## Introduction

Pulmonary toxicity secondary to antineoplastic agents is a well-known phenomenon, It is prevalent among at least 10% of patients receiving chemotherapy, and it results in significant morbidity and mortality [1]. There have been various studies that described drug-induced interstitial lung disease (DILD) and chemotherapy-related interstitial pneumonitis. Diagnosis of DILD is often made by exclusion. Pulmonary toxicity was frequently described in association with chemotherapy agents like bleomycin, methotrexate, Mitomycin-C, and carmustin [2]. But in recent times oxaliplatin is being identified as one of the drugs causing DILD. Oxaliplatin is well-known as a first-line chemotherapy agent in patients with gastrointestinal malignancies and it is usually used in combination with leucovorin (folinic acid) and 5-fluorouracil as FOLFOX regimen [3]. However, oxaliplatin-induced pulmonary toxicity is not a well-known side effect. This case report highlights the importance of identifying this rare but potentially life-threatening complication of oxaliplatin therapy. It demonstrates the need for early identification and treatment to prevent progression to pulmonary fibrosis and reversible lung damage.

## Case presentation

A 57-year-old African male was sent to the emergency department (ED) from the pulmonary clinic due to worsening hypoxia. He had stage IV gastric adenocarcinoma with metastasis to the liver on the FOLFOX chemotherapy regimen. Additional history of chronic smoking, hypertension, and hyperlipidemia was present. A review of his medical record indicated multiple recurrent admissions to the hospital for episodes of “shortness of breath and pneumonia” that had been managed with courses of steroids and antibiotics over the preceding 1 year. Furthermore, supplemental home oxygen therapy was prescribed at one of those visits.

On this presentation to ED, he was afebrile, hemodynamically stable, and found to be saturating 85 to 87% on his baseline home oxygen requirements of 4 L. Physical examination revealed a cachectic male with clear lung sounds. The rest of his physical exam was within normal limits. He was noted with elevated D-dimer and a computerized tomography (CT) chest with contrast was done to rule out a pulmonary embolism (PE). The results revealed severe chronic lung disease changes with no evidence of PE. He was started on IV methylprednisolone for presumed chronic obstructive pulmonary disease (COPD) exacerbation and admitted to the ward. A pulmonary consultation service was requested for the changes noted on the CT chest.

**Figure 1:**
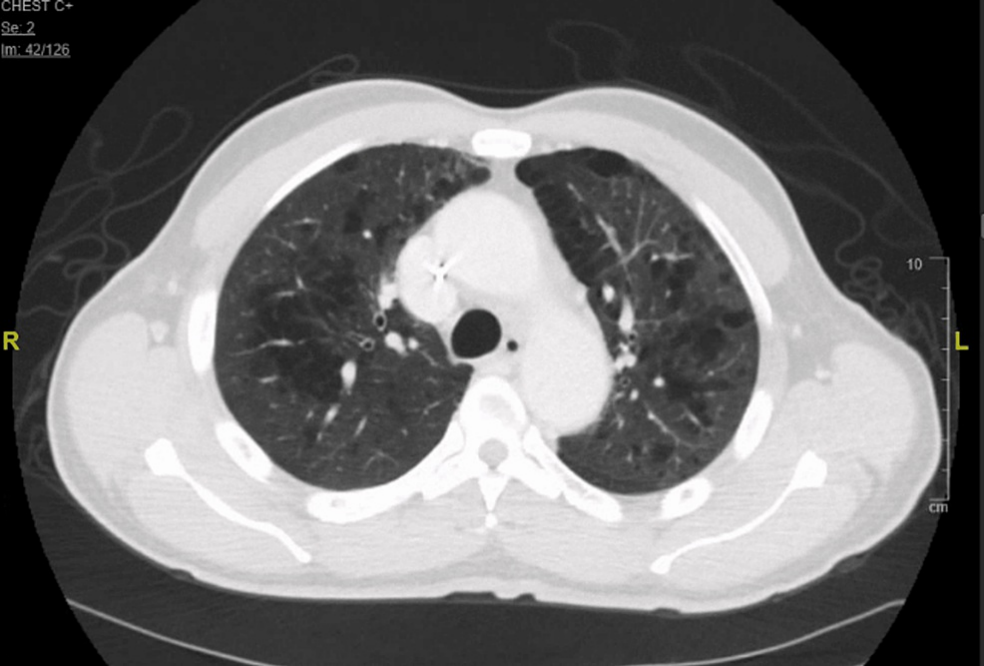
Initial CT chest prior to initiation of chemotherapy showing emphysematous changes

Upon review of his medical record, a staging CT chest performed 12 months prior to this admission demonstrated mostly normal lung parenchyma with minor emphysematous changes (Figure 1).

**Figure 2:**
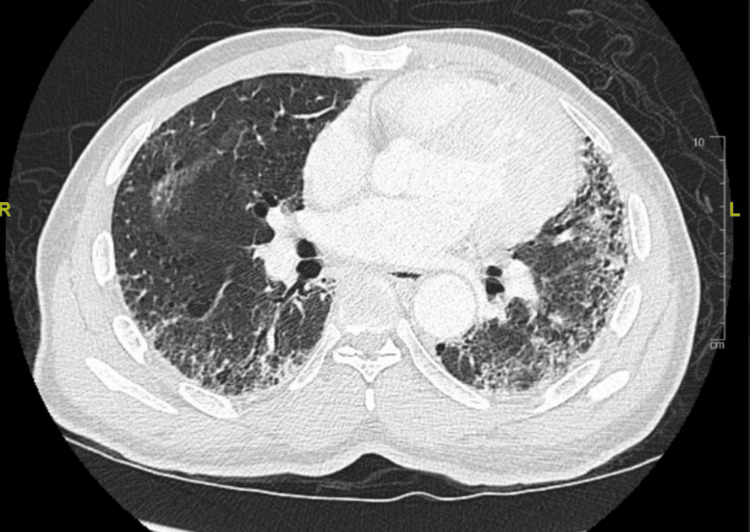
CT chest 3 months after chemotherapy showing changes suggesting pulmonary fibrosis

His spirometry was normal at baseline. Chemotherapy with FOLFOX was initiated. Three months into his chemotherapy, the patient required admission with worsening shortness of breath. CT chest done at that time (Figure 2) showed new fibrotic changes with honeycombing predominantly seen in the bases of the lungs.

**Figure 3:**
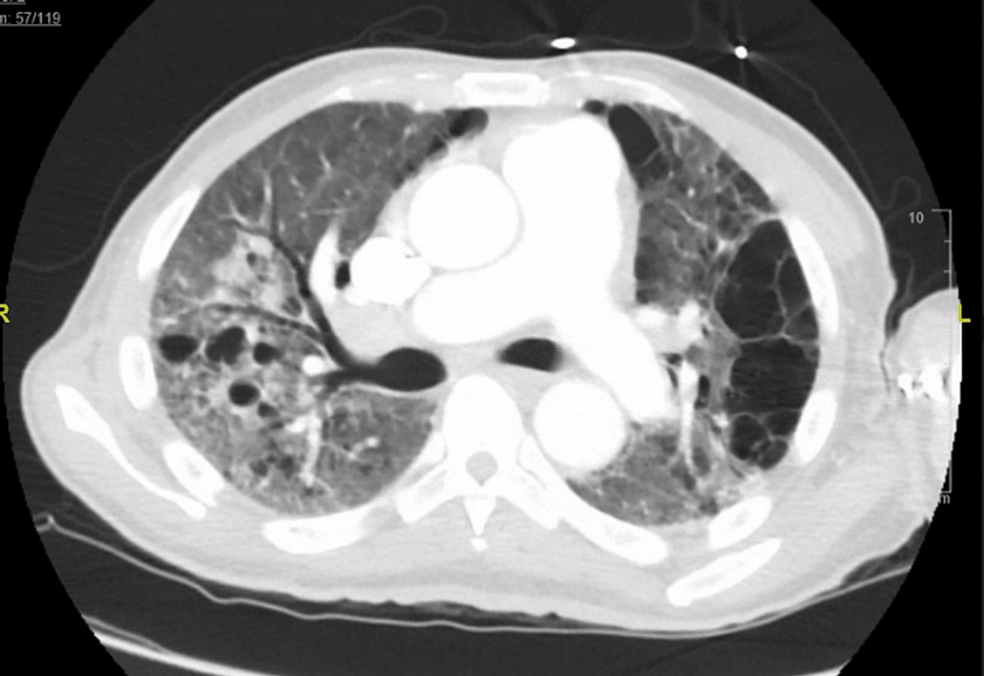
CT chest 1 year after chemotherapy showing advanced changes of honeycombing and traction bronchiectasis suggesting pulmonary fibrosis

**Figure 4:**
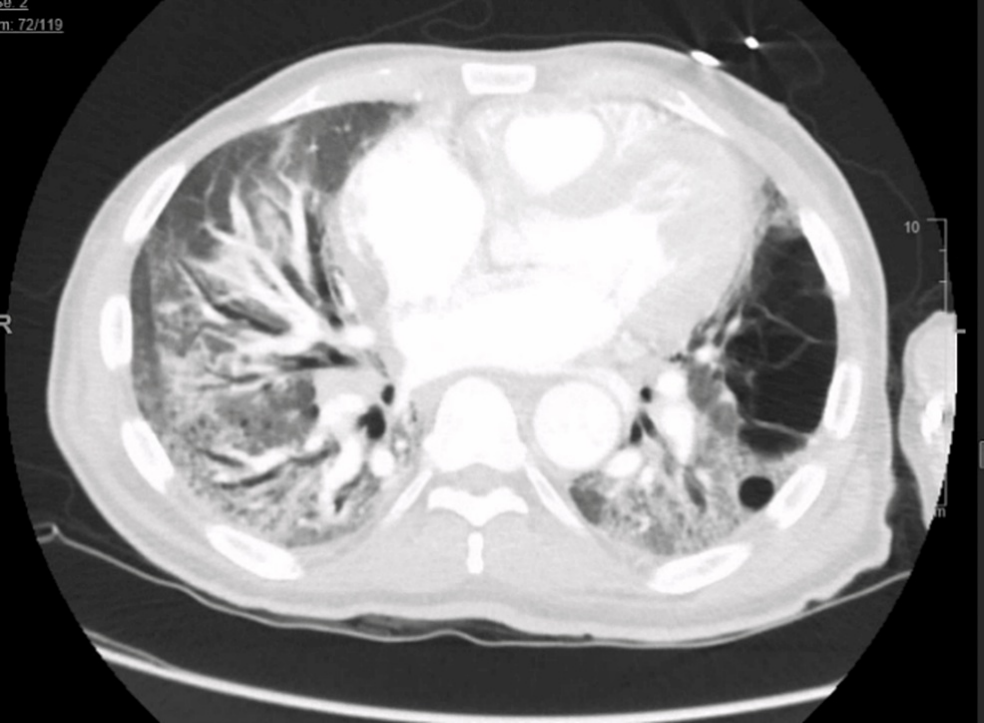
CT chest 1 year after chemotherapy showing advanced changes of honeycombing and traction bronchiectasis suggesting grade 4 pulmonary fibrosis

After discharge from the hospital, the patient’s oncologist stopped oxaliplatin due to the worsening respiratory symptoms, and the chemotherapy regimen was changed to 5-fluorouracil maintenance therapy without oxaliplatin. Subsequently, the patient was admitted to another hospital with “pneumonia” requiring mechanical ventilation, and again received steroids and antibiotics. Upon return to the oncology clinic a month later, his imaging (Figures 3, 4) showed an increase in the size of the metastatic lesions in the liver, after which the chemotherapy regimen was changed back to FOLFOX-based therapy with the addition of nivolumab.

About 2 months into his new chemotherapy cycle, he was seen in the pulmonary clinic. A review of his radiological studies over the course of oncological treatment suggested a temporal association between oxaliplatin therapy and worsening radiological pulmonary changes. CT chest progression revealed ground glass changes followed by gradual addition of fibrotic changes and culminating in full advanced fibrotic lung disease (Figures 1-4). Unfortunately, due to worsening hypoxia, he required another hospitalization. Eventually, after a discussion with oncology, his chemotherapy was held. In the subsequent oncology visit, his chemotherapy regimen was switched to FOLFIRI (leucovorin, fluorouracil, irinotecan). The patient was planned to follow-up with the pulmonary clinic and workup for other causes of ILD, such as connective tissue diseases, and to repeat imaging. But unfortunately, the patient was lost to follow-up, which was a limitation for a full ILD workup in this case. The patient had symptomatic improvement with antibiotics and steroids and bronchoscopy/ biopsy was deferred.

## Discussion

Oxaliplatin is commonly used in chemotherapy regimens for gastrointestinal malignancies in combination with leucovorin and fluorouracil as adjunctive therapy, well-known as the FOLFOX regimen [3]. Commonly identified side effects of oxaliplatin include gastrointestinal symptoms, peripheral neuropathy, hematologic and renal insufficiency [4]. Interstitial pneumonitis secondary to FOLFOX therapy is a rare disease. In trials that included oxaliplatin, less than 1% of patients were reported to have pulmonary fibrosis and grade IV pulmonary toxicity [4]. Recently more and more case reports show that oxaliplatin is being identified as a cause of DILD. There have been case reports regarding ILD secondary to FOLFOX therapy across the globe.

Most of the time, multiple antineoplastic agents are used together for chemotherapy regimen which makes it challenging to identify the culprit agents that cause a DILD. Nevertheless, discerning potential causes based on clinical evolution and previous case reports remains pivotal. At the time of this case report, there are no known cases of DILD caused by 5-fluorouracil or leucovorin except in situations where they were used in combination with other antineoplastic agents or radiation therapy. Muneoka et al. reported a patient with interstitial pneumonitis due to FOLFOX which resolved with cessation of oxaliplatin and did not recur when the patient was started on 5-fluorouracil and leucovorin without the oxaliplatin [5]. Another case report by Yague et al. reported DILD after chemotherapy with oxaliplatin alone [6]. Hence, the prevailing cause of FOLFOX-regimen-induced interstitial pneumonitis is primarily attributed to oxaliplatin. It is plausible that DILD due to oxaliplatin is underreported, its incidence could be higher than reported in the literature. Shimura et al. reviewed 734 patients who received FOLFOX or FOLFIRI, at least 11 patients had interstitial pneumonitis and all of these patients received FOLFOX therapy, in this study a higher incidence of DILD was noted at 1.5% compared to FDA-reported complications with oxaliplatin at 0.4% [1].

It is shown that preexisting lung conditions such as pulmonary fibrosis can lead to worsening DILD [7]. Hence it is pivotal that patients with underlying lung conditions should be monitored closely with frequent imaging when started on oxaliplatin therapy [3]. The most common symptoms of DILD include shortness of breath, cough, pleuritic chest pain, and less commonly fever [8]. The pathophysiology of oxaliplatin-induced pulmonary toxicity is unclear, it could be secondary to depletion of glutathione, this hypothesis was extrapolated from a study that showed liver damage with oxaliplatin [9]. Imaging modalities such as CT scans of the chest can help identify DILD, most frequently noted findings include interstitial infiltrates and ground-glass opacities, however, these are nonspecific findings that can be caused due to several other lung conditions [10]. Histologic findings that are commonly seen are organizing pneumonia and diffuse alveolar damage patterns [11] 

When DILD is suspected, the mainstay of treatment includes supportive care however high-dose systemic corticosteroid steroids are used in severe cases with persistent hypoxia. However, the role of high-dose systemic corticosteroids in oxaliplatin-induced interstitial pneumonitis is unclear [6]. Even with the use of high-dose systemic corticosteroids the mortality in these cases is very high [12]. In a case series of oxaliplatin-induced fibrosis, 5 out of 13 patients who received high-dose systemic corticosteroids expired [13]. Another case series from 2017 with 45 cases of FOLFOX-induced DILD showed a clear male preponderance [14]. Out of these patients, 5 patients had pulmonary fibrosis which was similar to our patient. If patients had only mild symptoms, early identification of DILD and discontinuation of oxaliplatin resulted in 100% resolution of the symptoms. Among the patients that received high-dose systemic corticosteroids outcomes in females were better than males and only 1 patient out of the 5 with pulmonary fibrosis showed improvement. But when patients presented with severe symptoms like hypoxia and respiratory insufficiency the prognosis was very poor and despite conventional treatment with steroids, 76.9% of these patients died. Most of these patients required intubation and mechanical ventilation and for all the patients who survived mechanical ventilation, a full recovery was noted [14].

## Conclusions

In summary, oxaliplatin-induced pulmonary fibrosis is a rare condition that can lead to life-threatening complications. Vigilant monitoring of drug-induced pulmonary toxicity is recommended for patients undergoing oxaliplatin-based chemotherapy, especially in those with underlying lung conditions. It is highly probable that the actual incidence is greater than reported, and that the literature only includes a limited number of cases. Further research is warranted to understand the risk factors like age, gender, ethnic or geographical differences, possible genetic predisposition, and prognostic factors for DILD associated with oxaliplatin. 
